# Surface Functionalization of Halloysite Nanotubes with Poly(acrylic acid) and Evaluation of Their Hemostatic Activity

**DOI:** 10.1021/acsomega.6c05088

**Published:** 2026-10-10

**Authors:** Kader Deren, Ceren Keçeciler-Emir, Cem Özel, Rasim Alosmanov, Muhammet U. Kahveci, Tuǧrul Elverdi, Sevil Yücel

**Affiliations:** † Department of Bioengineering, Faculty of Chemical and Metallurgical Engineering, 52999 Yildiz Technical University, Esenler, Istanbul 34220, Türkiye; ‡ YTÜ, MÜ, IÜC Health Biotechnology Excellence Joint Research and Application Center, Esenler, Istanbul 34220, Türkiye; § Department of Chemistry, Baku State University, Baku AZ 1148, Azerbaijan; ∥ Department of Chemistry, Faculty of Science and Letters, Istanbul Technical University, Maslak, Sariyer, Istanbul 34469, Türkiye; ⊥ Department of Internal Medicine, Division of Hematology, Cerrahpaşa Faculty of Medicine, Istanbul University-Cerrahpaşa, Istanbul 34098, Türkiye

## Abstract

Clays are widely used functional materials in industrial production due to their low cost, diversity, and easy accessibility. Since clays generally exhibit limited compatibility with other molecules, surface functionalization is often employed to modify their properties and expand their potential applications, particularly in biomaterials research. In this study, the potential of halloysite nanotubes (HNTs) as hemostatic materials was investigated by modifying their surface properties via photoinduced polymerization of acrylic acid (AA). PAA was coated onto MAPTES-silanized HNT substrates to enrich functional groups and modify the surface properties of the clay. The polymerization process was conducted at different polymerization times and MAPTES ratios to modulate the overall organic coating content on the HNT substrate. The structure–property relationships of the synthesized composites were comprehensively characterized, and their *in vitro* coagulation and platelet-recovery behaviors were evaluated. The results revealed that PAA modification altered the coagulation behavior of HNTs, resulting in changes in PT and aPTT responses that depended on the modification conditions. Furthermore, PAA modification altered the extent of platelet depletion from the recoverable plasma fraction compared with unmodified HNTs. These findings suggest that PAA modification influences the interaction between HNTs and blood components and provides a tunable surface-modification strategy, suggesting that PAA modification can modulate HNT–blood component interactions. Further studies using whole-blood and *in vivo* models are required to establish the hemocompatibility and hemostatic performance of the modified materials.

## Graphical abstract

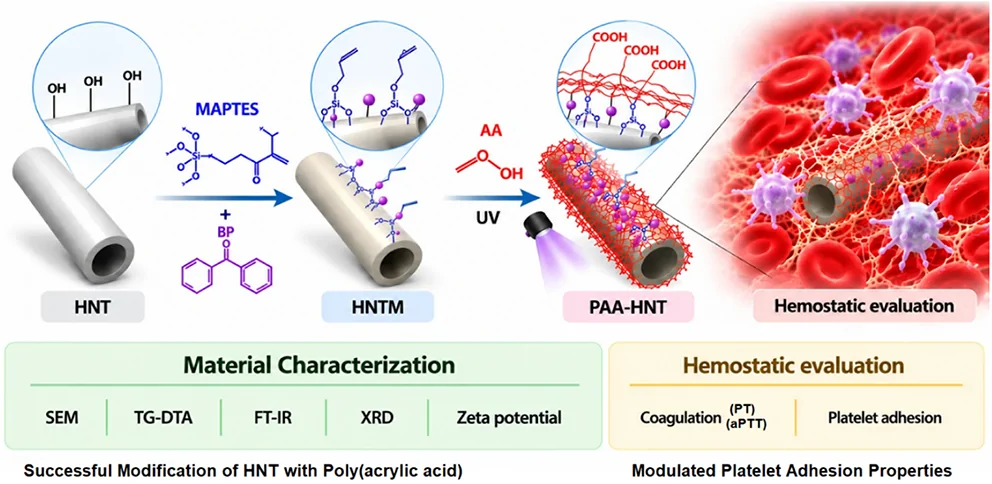

## Introduction

Clay is a versatile material that has been used since the dawn of human history and continues to find new applications over time. Beyond its abundance in global reserves and ease of access, the wide variety of clay types and mineral combinations within it has enabled its use in diverse fields.^1–3^ Due to its properties such as pH sensitivity, antimicrobial and antiviral behavior, biocompatibility, biodegradation, and bioinertness, it is used as an active ingredient, and due to its properties such as high swelling and absorption capacity and high surface area thanks to its porous structure, it is used as an excipient in the pharmaceutical, food, and textile industries.^4–9^

Halloysite nanotubes (HNTs) represent a class of aluminosilicate clays distinguished by their hollow, tubular nanostructure. Composed of alternating layers of aluminum oxide/hydroxide (inner layer) and tetrahedral silicate (outer layer), HNTs possess a high aspect ratio, a biocompatible nature, and abundant surface hydroxyl groups that enable facile chemical functionalization.^10,11^ Unlike conventional inorganic particles, HNTs exhibit distinct inner and outer surface chemistries, allowing for selective surface modification while preserving their intrinsic nanotubular architecture. Maintaining this native tubular morphology retains the mechanical stability and high specific surface area of HNTs, while the silanol-rich outer surface provides an effective platform for controlled surface functionalization.^10,12^

However, the limited interaction of clays with polymers and other molecules presents challenges such as low efficiency and premature degradation, particularly in drug delivery applications. To overcome these limitations, physical and chemical surface modifications have been employed to enhance interfacial interactions within the interlayers and on external surfaces. Recent studies have focused on functionalizing hydroxyl groups and surface defects located at the edges of clay structures.^13–16^

In polymer–clay composites, polymers typically serve as the matrix, while clays act as reinforcing additives. In this role, clays contribute to increased surface area, prolonged dissolution time, enhanced absorbency, improved gelation or structural thickening, and resistance to sedimentation.^17^ Photopolymerization applied to clay surfaces for different durations demonstrates that polymeric coating of varying compactness can be obtained. It is thought that clay surfaces functionalized would have a greater effect than pure clay, exhibit different properties in the biomedical field, and be applicable in various areas.^18–20^ Furthermore, the development of nanoscale composites has emerged as a key trend in this field, as nanomaterials provide a higher surface area-to-volume ratio, enabling stronger interactions and improved material performance.^21^ Many clay surfaces exhibit limited reactivity toward polymers. Therefore, studies suggest that modification using intermediate binders or matrix-forming agents prior to polymer incorporation leads to improved performance.^22,23^

Inorganic clay minerals, such as zeolite, kaolin, and halloysite nanotubes (HNTs), have various applications in different categories, including high fluid absorption, particle retention, antibacterial effects, and tissue regeneration. They are also extensively utilized in the literature as hemostatic agents or wound dressings.^8^ Since these materials are devoid of human or animal-derived proteins, they pose a negligible risk of allergic reactions or immunogenicity.^24–27^ Therefore, on the basis of their low immunogenicity and established efficacy, HNTs were selected as the primary inorganic component for the hemostatic agent. Beyond their inherent safety, HNTs hold significant potential for enhancing hemostatic properties given their proven efficacy in integrating functionalities into a composite material structure. It is generally accepted that synthetic polymers offer a more versatile platform than purely inorganic or biological counterparts, allowing for the precise engineering of hemostatic materials with tailored properties.^28^

Within the domain of synthetic polymers, polyacrylate-based polymers stand out because of their exceptional ease of manipulation and functionalization. The hemostatic activity of this class is often attributed to the electrostatic characteristics of the polymer chains, particularly cationic charges.^29^ For instance, amine-containing materials based on polyelectrolyte complex-coated poly­(lactic acid) and poly­(2-(dimethylamino)­ethyl methacrylate) have been observed to enhance red blood cell accumulation and coagulation.^30,31^ The function of such materials is governed by the electrostatic charge of their functional groups, a phenomenon further demonstrated by synthetic polymer hydrogels containing primary amines that induce factor VII activation.^32^ Additionally, poly­(2-hydroxyethyl methacrylate), a biocompatible and oxygen-permeable polyacrylate, is widely utilized in critical applications such as endovascular surgery.^33^ Drawing upon the proven versatility and charge-dependent efficacy of this polymer family, poly­(acrylic acid) (PAA) serves as the functional component to apply these surface modification strategies for enhanced hemostasis. PAA is a versatile and biocompatible polymer, whose carboxyl-containing structure can influence its interactions with plasma proteins and blood components. Its hemostatic activity is primarily driven by its negatively charged carboxyl groups, which trigger the coagulation cascade and accelerate the formation of thrombin in blood plasma. Previous studies have suggested that PAA-containing materials may modulate coagulation-related responses through surface charge, hydration, and interactions with plasma proteins; for instance, Forbes et al. reported that PAA-impregnated matrices reduced blood loss by 22% to 40%.^34^

This study investigates the surface modification of HNTs via photoinduced polymerization of acrylic acid to create a PAA coating which is chemically attached to HNTs for hemostatic applications. The primary aim was to prepare HNTs with different levels of PAA surface modification by varying the polymerization time (Figure 1) and to elucidate the resulting structure–property relationships. Second, the study comprehensively evaluates the potential of these PAA-modified HNT composites as novel hemostatic agents through *in vitro* characterization. This systematic investigation offers new insights into the relationship between surface modification parameters and the hemostatic performance of HNT-based composites.

**Figure 1:**
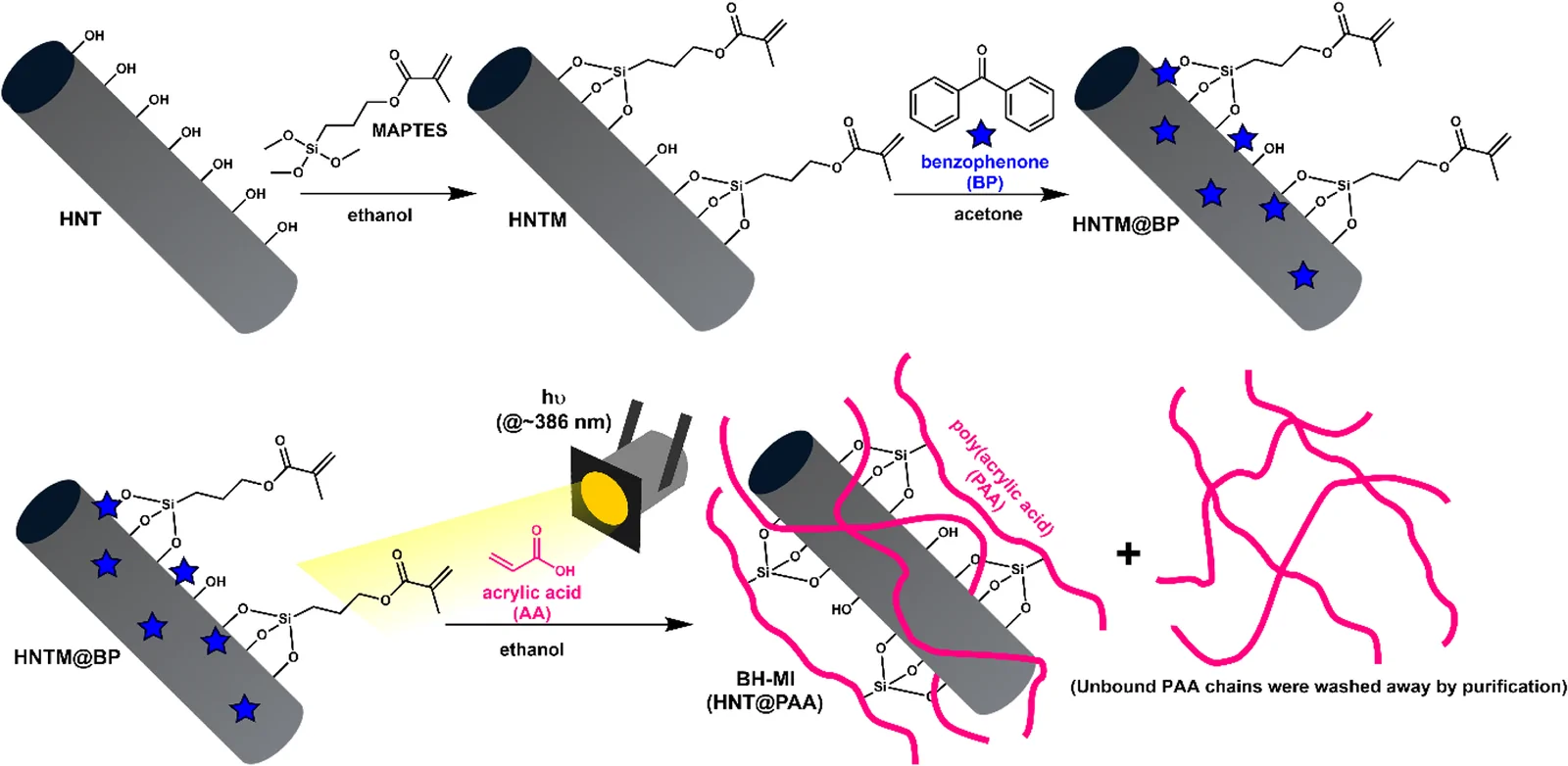
Surface modification of HNT with chemically bound PAA chains.

## Materials and Methods

### Materials

HNTs were purchased from Aldrich. Benzophenone (BP) and acrylic acid (AA) were purchased from Sigma-Aldrich (Steinheim, Germany). 3-(Trimethoxysilyl)­propyl methacrylate (MAPTES) was obtained from ABCR GmbH (Karlsruhe, Germany). Ethanol was supplied by ISOLAB Chemicals (Istanbul, Türkiye). The distilled water used during the experiments was water produced using a water distillation unit (GFL 2002, Germany). All chemicals were employed in their as-received states without further modification. AA was purified by vacuum distillation before the polymerizations.

### Surface Modification (Silanization) of HNTs

Surface modification of HNTs was performed via a silanization reaction with MAPTES. Briefly, HNTs were predried in a moisture analyzer at 105 °C for 5 min and then dispersed in a prehydrolyzed MAPTES/ethanol solution. MAPTES was added to the alcoholic solution at different ratios. Subsequently, the pH of the solution was adjusted to 4.5–5.0 using glacial acetic acid. The dispersion was subjected to a silanization reaction under reflux for 30 min.^35,36^ To prevent sedimentation and homogeneity problems during the reaction, a magnetic stirrer was placed under the reflux system. The stirrer was set to 500 rpm.

To optimize the coating efficiency, various mass-to-volume ratios of HNT (g) to MAPTES (mL) were mixed (1:5, 1:10, and 1:20), and the resulting samples were coded as HNTM 1:5, HNTM 1:10, and HNTM 1:20, respectively.

After the reaction, the MAPTES-HNTs (HNTM) were separated by centrifugation at 9000 rpm for 5 min. The pellets were washed with absolute ethanol (3 times) with to remove unreacted MAPTES. After each washing step, the HNTs were redispersed by gentle magnetic stirring for 5 min, followed by centrifugation under the same conditions. Finally, the pellets were dried in a vacuum oven at 55 °C for 24 h.^35,36^ Unmodified HNTs were used as a control group for comparative analysis.

### Immobilization of the Photoinitiator on HNTMs and Polymerization

Prior to polymerization, the BP photoinitiator was physically immobilized onto the MAPTES-silanized HNT (HNTM) surfaces. A BP stock solution (10 g/L) was prepared by dissolving 50 mg of BP in 5 mL of acetone within a reaction tube wrapped in aluminum foil to prevent photoactivation. The immobilization process was conducted on a shaker for 1 h under an inert gas atmosphere, and 300 mg of HNTMs immobilized by BP was dried in ambient conditions. Following immobilization, the mixture was homogenized with the addition of ethanol.

To extend its shelf life, commercially available acrylic acid (AA) contains the polymerization inhibitor 4-methoxyphenol (MEHQ). Since MEHQ may reduce polymerization efficiency, it was removed prior to use by vacuum distillation under a reflux system. During the distillation process, the condenser temperature was maintained at +8 °C. The distillation temperature was gradually increased from 75 to 113 °C to ensure rapid evaporation of AA while minimizing premature polymerization. The distilled AA vapor was condensed at +8 °C and collected in an amber glass bottle. To minimize oxidation, the purified monomer was purged with an inert gas, tightly sealed, and stored in a light-protected environment until use.

Following immobilization, the mixture was homogenized with the addition of ethanol. Subsequently, a pure AA monomer was introduced into the suspension at 1 mL volumes, and the system was purged with inert gas to remove dissolved oxygen. The reaction tube was then placed in a photoreactor. The distance between the UV lamp (photoreactor equipped with 6 cylindrical bulbs emitting light at around 366 nm with an average light intensity of 50 mW/cm^2^ at the location of polymerization) and the reaction tube was positioned as 10 cm. UV-induced polymerization onto HNTs was carried out for varying durations: 1 h (BH-MI 1 h), 2 h (BH-MI 2 h), and 24 h (BH-MI 24 h). For the control group, polymerization onto HNTs was carried out without BP immobilization (BH-M 1:5 24 h). Because the polymerization was performed under free-radical conditions without direct control or measurement of the molecular weight of the grafted PAA, variation in polymerization time was considered as a parameter affecting the overall extent of surface polymerization rather than as an isolated control of the grafting density or polymer chain length.

To ensure a homogeneous reaction and prevent sedimentation of clay nanoparticles, continuous stirring at 1400 rpm was maintained throughout the process. Upon completion of the reaction, the resulting composites were washed repeatedly with ethanol to remove unreacted monomers and finally dried in a vacuum oven at 55 °C for 24 h.

### Characterization of the Samples

The chemical structures and functional groups of the modified HNTs and synthesized polymeric composites were analyzed using Fourier Transform Infrared Spectroscopy (FT-IR). The spectra were recorded using a Shimadzu IRPrestige-21 spectrometer (Shimadzu Corp., Japan) equipped with an Attenuated Total Reflection (ATR) module. Measurements were performed over a spectral range of 4000 to 650 cm^–1^.

The crystal structure changes of all samples before and after surface modification were investigated using X-ray diffractometry (XRD). The diffraction patterns were recorded in the 2θ scanning range of 5–65 °

The zeta potential (ζ potential) of the samples was measured at 25 °C using a 4.0 mW He–Ne laser (633 nm) and a 90° scattering angle with a Zetasizer Nano ZS device (Malvern Instruments, Worcestershire, UK). The zeta potential distribution was determined using the Laser Doppler Electrophoresis method (n = 3).

The morphological properties (surface and size) of the samples were characterized using a scanning electron microscope (SEM; Zeiss EVO LS 10 T). To prevent surface charging and improve image quality, the samples were sputter-coated with gold–palladium prior to analysis. The coating was applied using an Emitech K550X device (UK) under vacuum.

The thermal behavior and organic content of the HNTMs and PAA-coated HNTs were comprehensively investigated using simultaneous thermogravimetric and differential thermal analysis (TG/DTA). Measurements were conducted using a TG/DTA (Seiko Instruments SII, Exstar TG/DTA 6300) system. Samples were heated in ceramic crucibles from 30 to 1000 °C at a constant heating rate under a dry air atmosphere. To evaluate the decomposition stages and thermal transitions, mass loss (TGA), derivative thermogravimetry (DTG) for determining maximum degradation rates, and differential thermal analysis (DTA) for identifying endo-/exothermic phase transitions were concurrently recorded.^36,37^

### Evaluation of the Hemostatic Activity of Composites

Pooled plasma, which was obtained from 20 healthy donors with PT and aPTT values within the normal reference range, was prepared from citrated blood samples (centrifuged at 3500 rpm for 10 min), with Prothrombin Time (PT) (12 s, 76.4% activity) and activated partial thromboplastin time (aPTT) (25 s) values verified within normal clinical limits. Control blood samples were selected from among those with a normal hemogram and PT-aPTT values. Citrated blood samples were obtained from volunteer donors. After confirming that the blood counts (platelet counts) in the collected samples were normal, platelet-rich plasma (PRP) was obtained from a portion of these blood samples by centrifuging at 800 rpm for 10 min. The pooled PRP sample was counted using an automatic blood cell counter, revealing a platelet count of 388,000/μL. After obtaining platelet-poor plasma (PPP), a separate pooled sample was created from the erythrocyte suspension remaining at the bottom to obtain pure erythrocytes.

Samples were prepared as suspensions by dispersing them in phosphate-buffered saline (PBS) at a concentration of 0.2 g/mL, in accordance with the ISO 10993-12:2012 standard for sample preparation and reference materials.^38^ The suspensions were prepared by adding 5 mL of PBS to 1 g of sample.^39^ The study protocol was approved by the Clinical Research Ethics Committee of Istanbul University-Cerrahpaşa (date: 29/07/2020, No. 98599).

#### In Vitro Blood Clotting Tests

To evaluate the PT and aPTT, samples of 5 μL volume were introduced into Eppendorf tubes containing 200 μL of PPP. Following brief agitation at low speed, the mixtures were incubated at 37 °C for 5 min. PT measurements were conducted using a Siemens BCS XP automated coagulometer. Unmodified HNTs were utilized as reference materials for these experiments.^39^

### Platelet Adhesion Test

Platelet adhesion and aggregation potential were evaluated by introducing 150 μL of PRP into Eppendorf tubes containing the composites. The samples were incubated at 37 °C for 30 min without agitation to preclude spontaneous platelet activation. Platelet concentrations were determined using a Beckman Coulter automated hematology analyzer. The platelet count of the pooled PRP before contact with the material was used as the baseline reference value (n_0_),^39^ and the platelet count after incubation was recorded as *n*_*t*._ Platelet depletion was calculated as shown in eq 1:

1 $$Platelet\;adhesion\;ratio\;\left(\%\right)=\frac{n_{0}-n_{t}}{n_{0}}\times 100$$

This assay measures the reduction in recoverable platelet count after material contact. In a subsequent series of experiments, mixtures containing equal volumes (100 μL each) of PRP and composite were analyzed at 0 and 30 min to monitor temporal changes in platelet count. All experiments were performed in triplicate, and the data were analyzed using one-way ANOVA.

## Results and Discussion

In the current study, HNTs were chemically modified with PAA chains through photoinduced polymerization mediated by the benzophenone (BP) photoinitiator (Figure 1). For this purpose, first, HNTs were modified with MAPTES by typical silanization. Then, BP was physically adsorbed onto the surfaces of the MAPTES-silanized HNT (HNTM) substrate by simple mixing (HNTM@BP). Eventually, acrylic acid (AA) was induced upon irradiation of a colloidal mixture of AA and HNTM substrates containing BP. The copolymerization of methacrylate moieties of the MAPTES groups attached to the HNT substrates with AA yielded PAA chains chemically bound to the HNT substrates (BH-MI). Meanwhile, nongrafted polymer chains may also have formed during the reaction and were removed as far as possible through repeated washing steps.

The experimental design comprised two sequential optimization series. First, the MAPTES/HNT ratio was varied (1:5, 1:10, and 1:20) to evaluate its effect on surface functionalization and the resulting organic coating. Subsequently, the MAPTES 1:5 ratio was selected as the baseline formulation for the systematic evaluation of the PAA polymerization time (1, 2, and 24 h) with and without immobilization of BP. Accordingly, physicochemical characterization techniques were used comparatively within these optimization series, whereas the subsequent *in vitro* hemostatic evaluation focused on the selected 1:5 series.

### FT-IR

The FT-IR spectrum and corresponding characteristic absorption bands of the samples are presented in Figures 2 and 3. The bands around 3695 and 3621 cm^–1^ are attributed to the stretching vibrations of the −OH groups present within (internal) and on the surface of pure HNT.^35,40^ Strong absorption bands associated with asymmetric stretching vibrations of Si–O–Si and Si–O–Al in the region from ∼1150 down to 500 cm^–1^ confirm the aluminosilicate structure of HNTs. Specifically, the peaks within this range, in descending order, correspond to the apical Si–O in-phase stretching at 1122 cm^–1^; the Si–O in-plane stretching vibration at 1005 cm^–1^; the Al–OH bending vibration at 901 cm^–1^; the Si–O vibrations at 788, 751, and 663 cm^–1^; and, finally, the Al–O–Si and Si–O–Si bending vibrations at 596 cm^–1^, respectively.^41–44^ After HNTs were modified with a silane agent, several new bands were observed in the FT-IR spectra. HNTM (1:x) denotes HNTs whose surfaces were silanized using MAPTES at varying HNT/MAPTES ratiosWhen comparing 1:5, 1:10, and 1:20 HNTM with pure HNT, stretching vibrations of aliphatic −CH_2_ and −CH_3_ groups belonging to the MAPTES silane agent were observed as new peaks at 2970 and 2829 cm^–1^.^43^ C=O and C=C stretching vibrations at 1718 cm^–1^, C=C stretching vibrations associated with the methacrylate ester group at 1638 cm^–1^, the deformation of the Si–CH_2_ bond at 1263 cm^–1^, 1189 cm^–1^ for the C–O–C ester bond in MAPTES, and 1156 cm^–1^ for the bond belonging to the −Si–O–Si– group in MAPTES were observed.^45,46^

**Figure 2:**
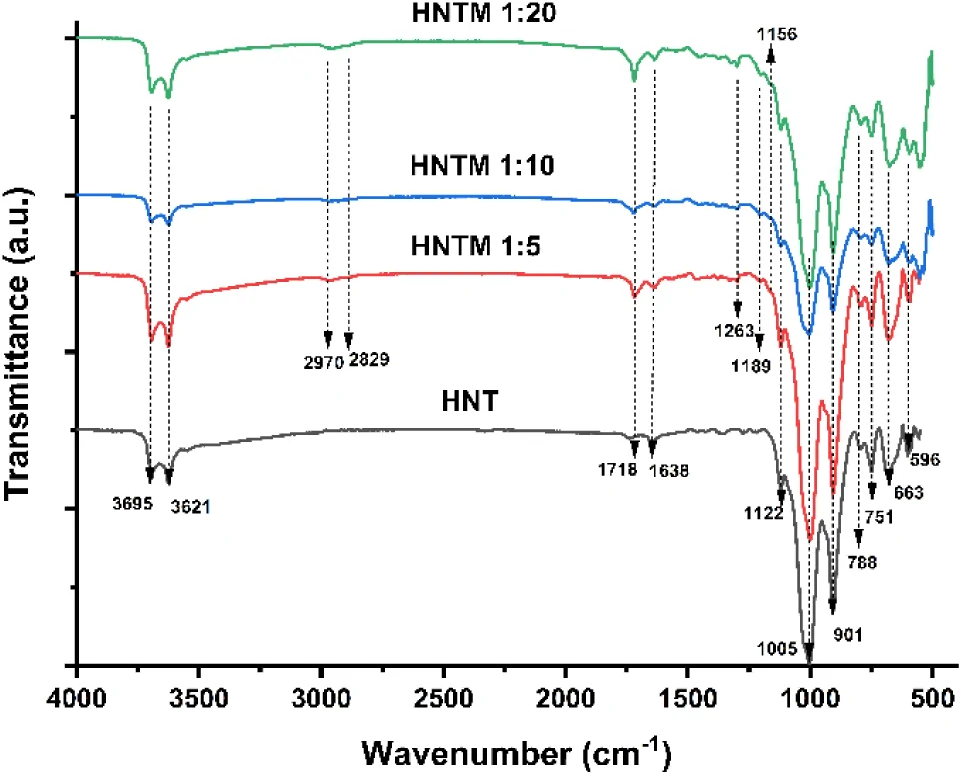
FT-IR spectra of HNT and HNTMs.

**Figure 3:**
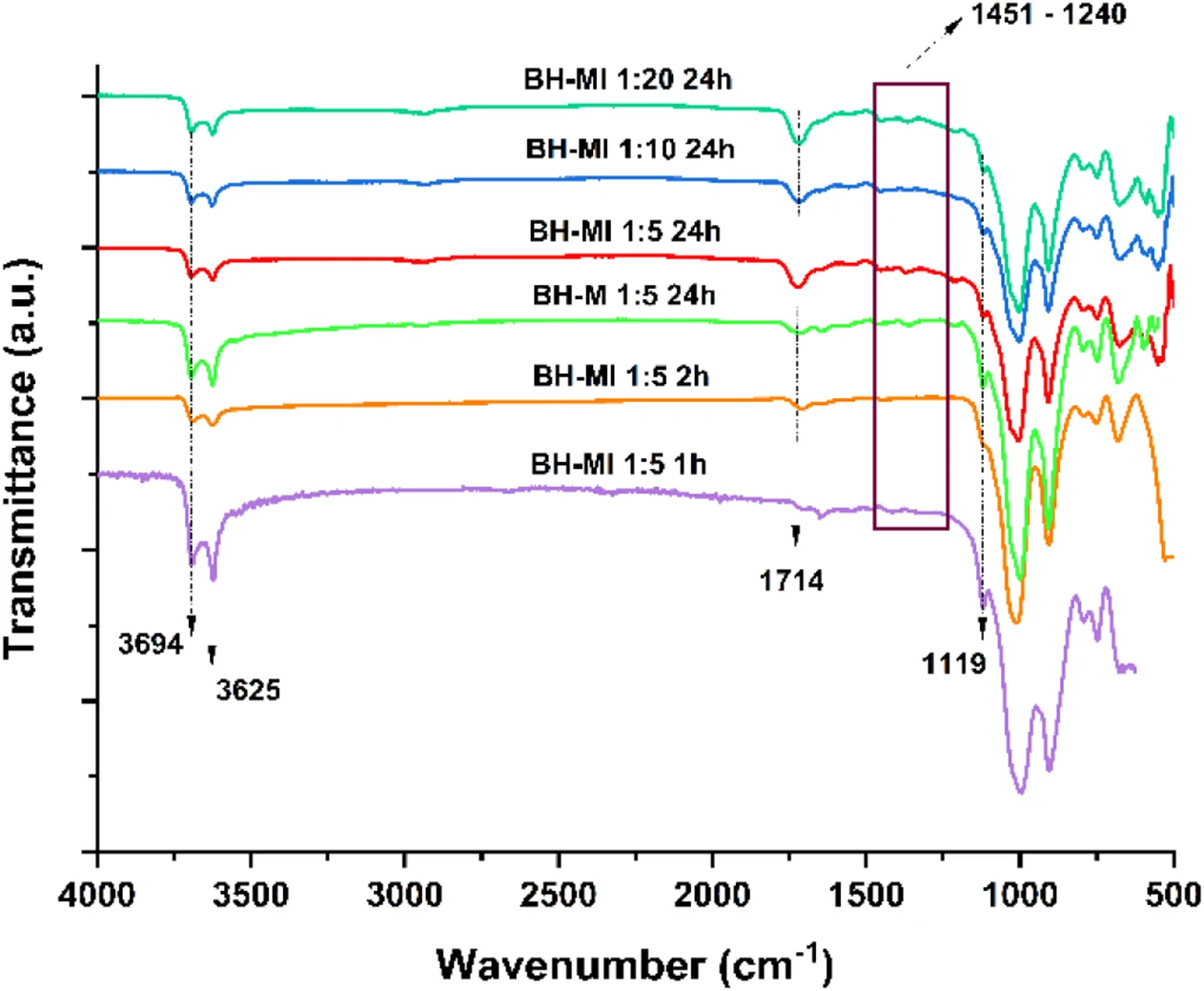
FT-IR spectra of BH-MIs.

Figure 3 shows the FT-IR spectrum corresponding to MAPTES-silanized halloysite nanotubes on which PAA chains were ungrafted (BH-M) or grafted through a benzophenone-initiated polymerization process (BH-MI), expected to yield PAA chains covalently anchored through the surface-bound methacrylate groups. Since both the propyl groups of MAPTES and the grafted PAA chains contain aliphatic methylene groups, this band may receive contributions from both components and therefore should not be considered as direct evidence of PAA grafting alone. The absorption band in the 1714 cm^–1^ region is associated with C=O stretching vibrations and is already present in the MAPTES-silanized HNTs (Figure 2), indicating that this region cannot be used as a standalone diagnostic band for PAA grafting (Figure 3). After polymerization, changes in the intensity and profile of this band may partly arise from the overlapping contributions of the MAPTES methacrylate ester and PAA carboxylic acid groups.^48,49^ The peaks observed at 1714 and 1451 cm^–1^ are associated with symmetric and asymmetric stretching vibrations of COO^–^ groups, respectively.^37,48^ The band around 1451 cm^–1^ is assigned to CH_2_ deformation, while the bands around 1412 and 1240 cm^–1^ can be assigned to C–O stretching coupled with O–H in-plane bending vibrations.^47^ The increased intensity of 1240–1119 cm^–1^ bands with prolonged polymerization time is consistent with changes in the overall organic coating on the HNT surface; however, FT-IR alone cannot distinguish changes in grafting density from changes in polymer chain length. Within this region, the peak at 1119 cm^–1^ specifically corresponds to the CH_2_ bending vibration. In combination with the washing procedure and the complementary TGA results, these spectral changes support the retention of a stable PAA-containing organic layer on the HNT surface;^50^ however, FT-IR alone does not provide definitive evidence to distinguish covalently grafted PAA from strongly retained physically associated polymer.

The characteristic clay-related bands in the 3695–3621 cm^–1^ and 1008–909 cm^–1^ regions exhibited slight changes in position and intensity after polymerization. These changes may reflect modifications in the local chemical environment of the HNT surface hydroxyl and aluminosilicate groups following formation of the organic coating; however, such small spectral changes alone are not considered sufficient evidence for PAA grafting. Furthermore, the characteristic HNT bands remained evident after silanization and polymerization, suggesting that the fundamental aluminosilicate framework was retained during the surface modification process.^41^ The appearance of new organic bands and the increased intensity of characteristic organic absorptions, together with the complementary TGA results, support the formation of MAPTES- and PAA-containing organic layers on the HNT surface. The increased intensity observed at higher MAPTES/HNT ratios is consistent with an increased overall organic coating content; however, it does not by itself distinguish between changes in grafting density and polymer chain characteristics.

### Zeta Potential

Table 1 presents the results of the average surface charges for HNTs, HNTMs, and BH-MI composites. In the study conducted on HNTs, which have a negative charge on the outer surface and a positive charge on the inner surface,^51^ the zeta (ζ) potential was measured as −35.25 mV due to the dense OH groups on the surface. HNTMs after silanization showed a similar ζ-potential with an average of −29 mV. Following silanization, the ζ-potential values became less negative, indicating changes in the surface chemistry. The BH-MI samples also exhibited less negative ζ-potentials compared with pristine HNTs; however, the values did not show a monotonic variation with polymerization time or MAPTES ratio. The lowest value measured was −22.9 mV, as expected. The negative charge decreased due to the reduction in free OH groups on the clay surface because of MAPTES and PAA binding to the hydroxyl groups present on the surface.^52^ This behavior indicates that the measured ζ-potential is influenced not only by the surface functional groups but also by the structure, coverage, hydration, and organization of the polymer layer.

**Table 1:** Results of Zeta Potential, XRD, and Organic Coating Content Based on TGA of HNTs, HNTMs, and Polymer-Coated HNT (BH-MI)

	Surface charge	XRD				TGA
		d001 plane		d020 plane		
	Average zeta potential (mV)	2θ	Å	2θ	Å	Total mass loss (%)
**HNT**	–35.25	11.53	7.66	20.01	4.43	14.23
**HNTM** 1:5	–29.55	11.79	7.75	19.88	4.46	18.22
**HNTM** 1:10	–29.20	11.71	7.55	20.07	4.42	19.61
**HNTM** 1:20	–29.13	11.68	7.57	19.70	4.45	21.57
**BH-M** 1:5 **24 h**	–29.16	11.81	7.49	19.92	4.45	19.23
**BH-MI** 1:5 **1 h**	–29.73	11.41	7.74	19.92	4.45	20.88
**BH-MI** 1:5 **2 h**	–29.43	11.79	7.50	19.92	4.45	20.36
**BH-MI** 1:5 **24 h**	–24.56	11.56	7.64	20.21	4.39	24.83
**BH-MI** 1:10 **24 h**	–24.90	11.88	7.44	19.92	4.45	25.61
**BH-MI** 1:20 **24 h**	–22.90	11.92	7.41	19.87	4.46	28.11

Compared with pristine HNTs, the ζ-potential became less negative. This change is in good agreement with the FTIR analysis, which demonstrated the successful surface functionalization of HNTs through MAPTES silanization and subsequent PAA coating. Overall, the ζ-potential results, together with the FTIR findings, support the successful surface functionalization of HNTs through MAPTES silanization and subsequent PAA coating.

### XRD

The XRD diffractogram and basal spacing calculated by Bragg’s law for the samples are presented in Figures 4 and 5 and Table 1. Pure HNT d001 diffraction crystal peaks at 11.53° 2θ confirm the tubular structure of HNT clay with a 7.66 Å basal spacing, while d020 crystal peaks at 20.01° 2θ confirm a 4.43 Å basal spacing (Figures 4 and 5). In the literature, HNT clay has been reported to have a d001 crystal diffraction of 7.21–7.4 Å ^53,54^ and a d020 diffraction of 4.45 Å.^55^ The diffraction peak at 26.49° 2θ indicates the presence of quartz, while the diffraction peaks between 35° and 40° 2θ indicate a calcite structure.^56^ No significant changes were observed between pure HNT and the modified with MAPTES agent and PAA grafted onto HNTs, except for minor variations. This minor change could be explained as the continuous expulsion of water from the interlayers.^54^

**Figure 4:**
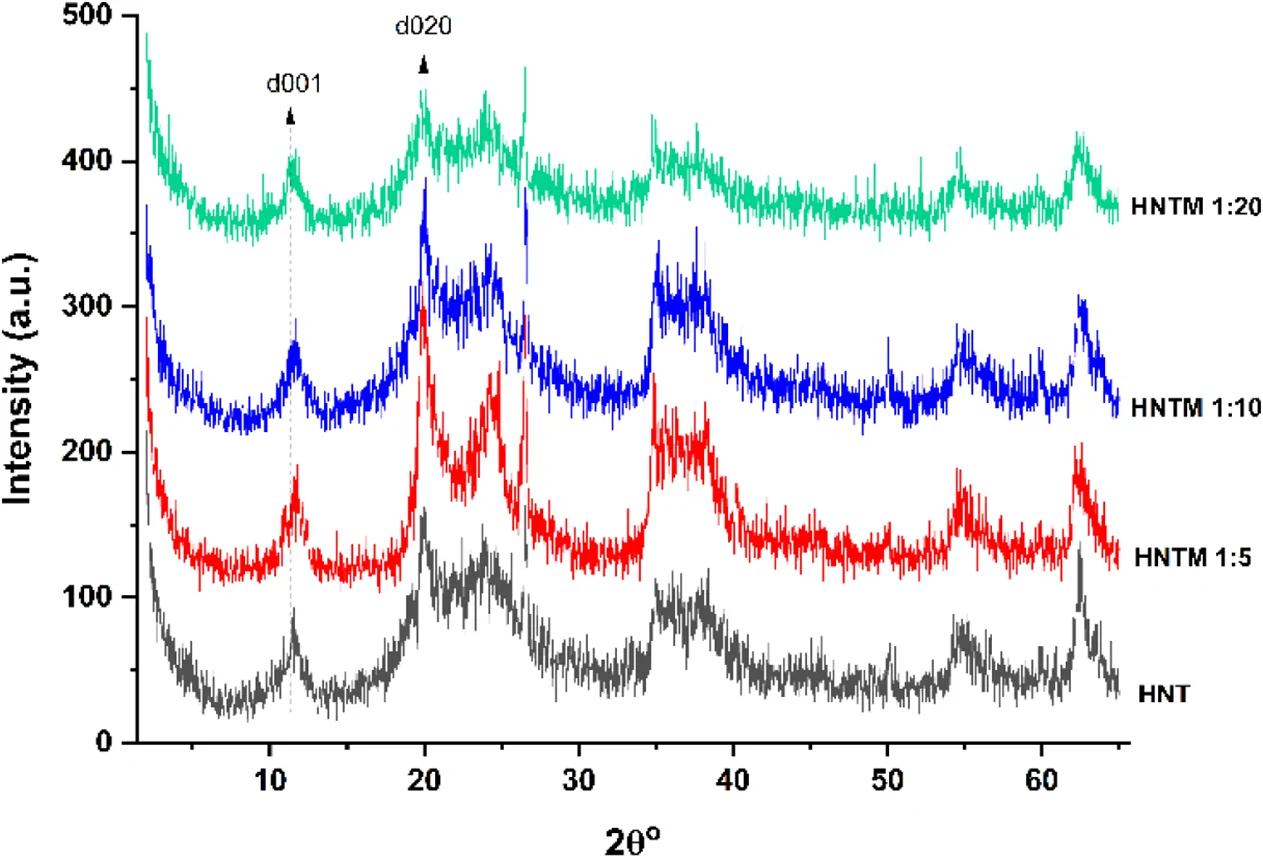
XRD spectra of HNT and HNTMs.

**Figure 5:**
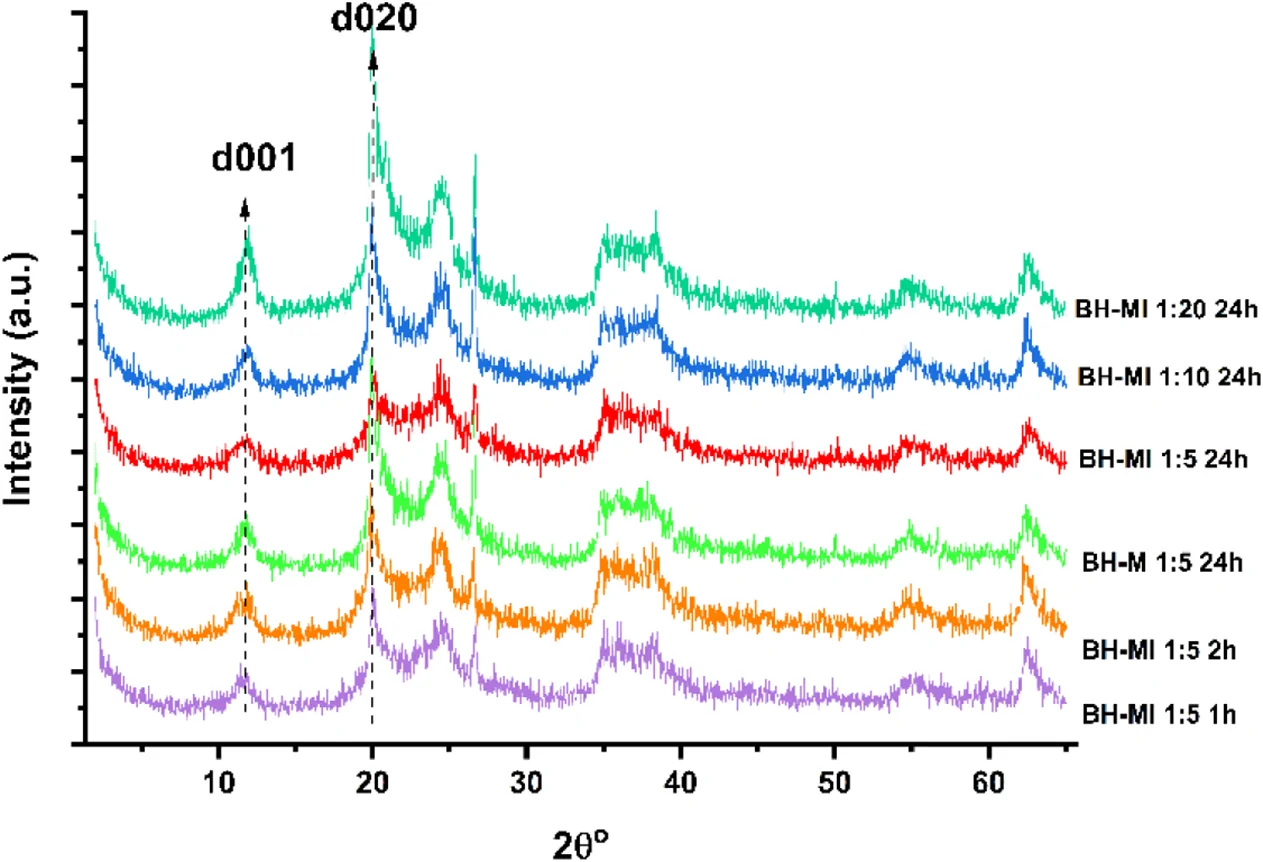
XRD spectra of BH-MIs.

An increase in the basal gap allows a molecule to bind between the layers of the HNTs. A partial decrease in the basal gap can cause molecules binding to the edges of the HNTs to create a contraction in the layers, leading to a further reduction in the basal gap. Therefore, no change may be observed between the layers of the HNTs. Provided that the effects of the reacting molecules are successfully demonstrated through other analyses, it can be said that binding occurs on the surface of the HNTs.^57,58^ The absence of any noticeable change in the basal spacings suggests that the silane coupling agent and consequently the PAA coating were predominantly associated with the outer surface of the HNTs rather than being intercalated into the inner lumen or interlayer spaces.

After silanization and polymerization, the crystalline structure of the HNTs remained intact, while the gradual removal of interlayer and adsorbed water during the modification process caused only minor variations in peak intensity and position.^41,53,58–61^ If intercalation had occurred, then a noticeable change in the basal spacing (d001) would be expected. However, the absence of significant changes in the basal spacing indicates that MAPTES and the grafted PAA predominantly occurred on the external surface of the HNT nanotubes rather than intercalated into the lumen or interlayer regions. This interpretation is consistent with the FTIR results, which confirmed successful MAPTES silanization and a subsequent PAA coating.

Together with the FTIR peaks confirming successful MAPTES silanization and subsequent PAA coating, XRD results demonstrate that surface functionalization was achieved while preserving the intrinsic crystalline structure of the HNT.

### SEM

According to the SEM images obtained, as expected, pure HNTs were observed to have tubelike shapes with very small particle sizes (Figure 6). Following MAPTES silanization and PAA modification, the modified HNT particles exhibited a greater tendency toward agglomeration compared with pristine HNTs.^42^ These morphological changes are consistent with the formation of an organic surface layer; however, SEM alone cannot confirm the presence or chemical nature of the polymer coating. As can be observed from the SEM images, the modified HNT particles exhibited a greater tendency to adhere to each other as they functionalized from silanization (Figure 6b) to coating (Figure 6c) compared to pure HNTs (Figure 6a); this could be consistent with increasing the formation of an organic coating.^62^ In addition, Zhang et al. report that the polymer coating is not expected to be clearly distinguished by SEM alone, as the high HNT content may limit the volumetric shrinkage of the polymer matrix during drying, thereby reducing the contrast of the coating in SEM micrographs.^63^ While there is no change in the tubular morphology as a result of silanization and polymerization, the absence of change in tubular diameters is consistent with the XRD analysis.

**Figure 6:**
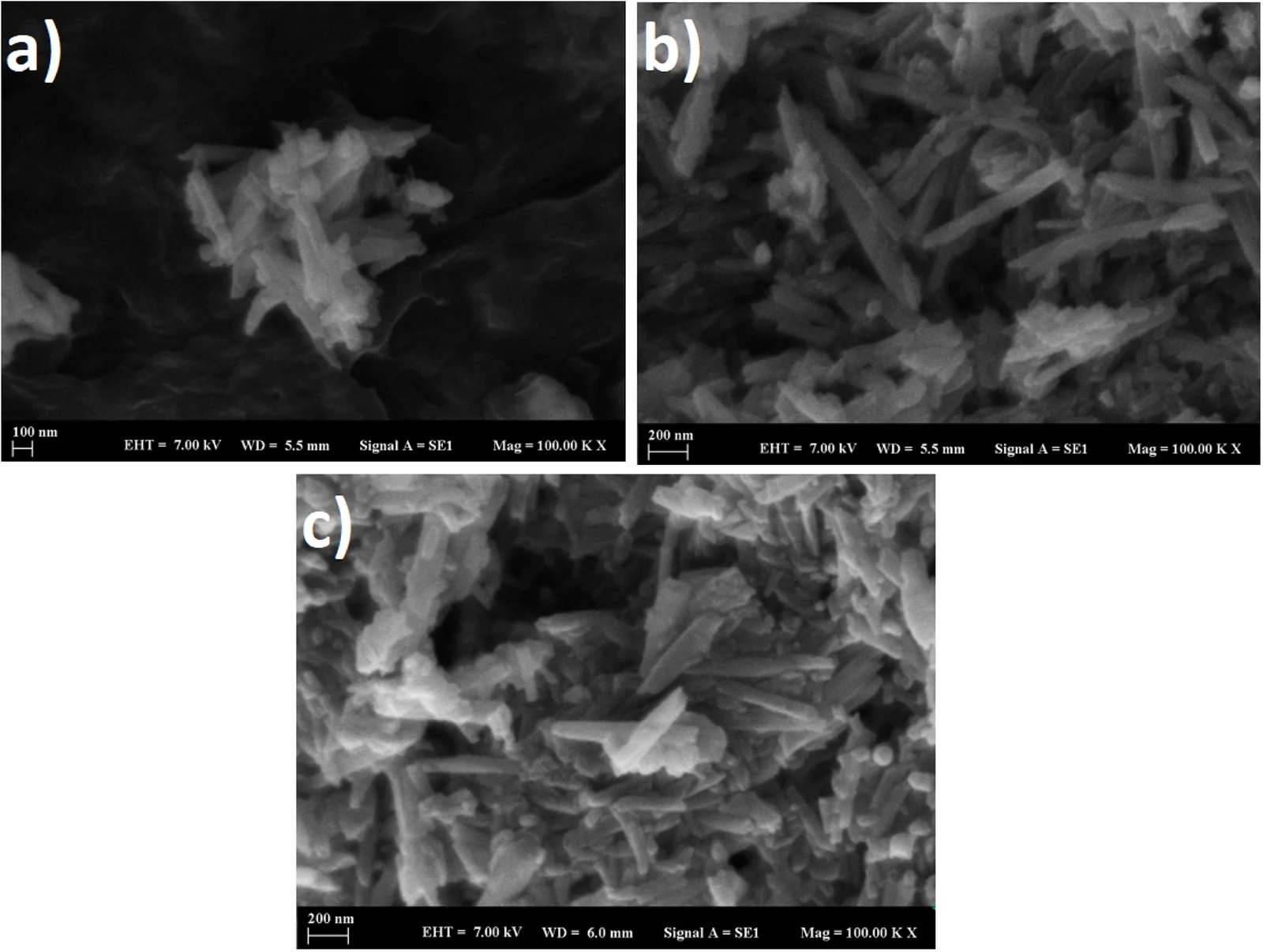
SEM images: a) HNT, b) HNTM, and c) BH-MI 1:5 24 h.

### Thermal Analysis

The thermal analysis results of the samples, comprising TGA (Figures 7 and 8), DTG (Figures 9 and 10), and DTA (Figures 11 and 12) profiles, are presented in Figures 7–12, and the corresponding quantitative thermal data are summarized in Table 1. As detailed in the table, the first stage of mass loss occurs below 100 °C and is attributed to the dehydration process. This step involves the elimination of free and physically bound water trapped within the nanotubular channels and exterior surfaces of the HNTs, confirming the high water-retention capacity of the prepared composites.^41,64^ In Figure 7, the relatively limited mass loss observed for pristine HNT between approximately 350 and 400 °C indicates that the major thermal degradation/dehydroxylation process had not yet occurred to a substantial extent within this range.^65^ In the temperature range of 500–1000 °C, approximately 70–85% of the mass is retained as inorganic clay residue.^66^ Variations in mass loss below 500 °C reflect degradation processes related to silanization and polymerization.

**Figure 7:**
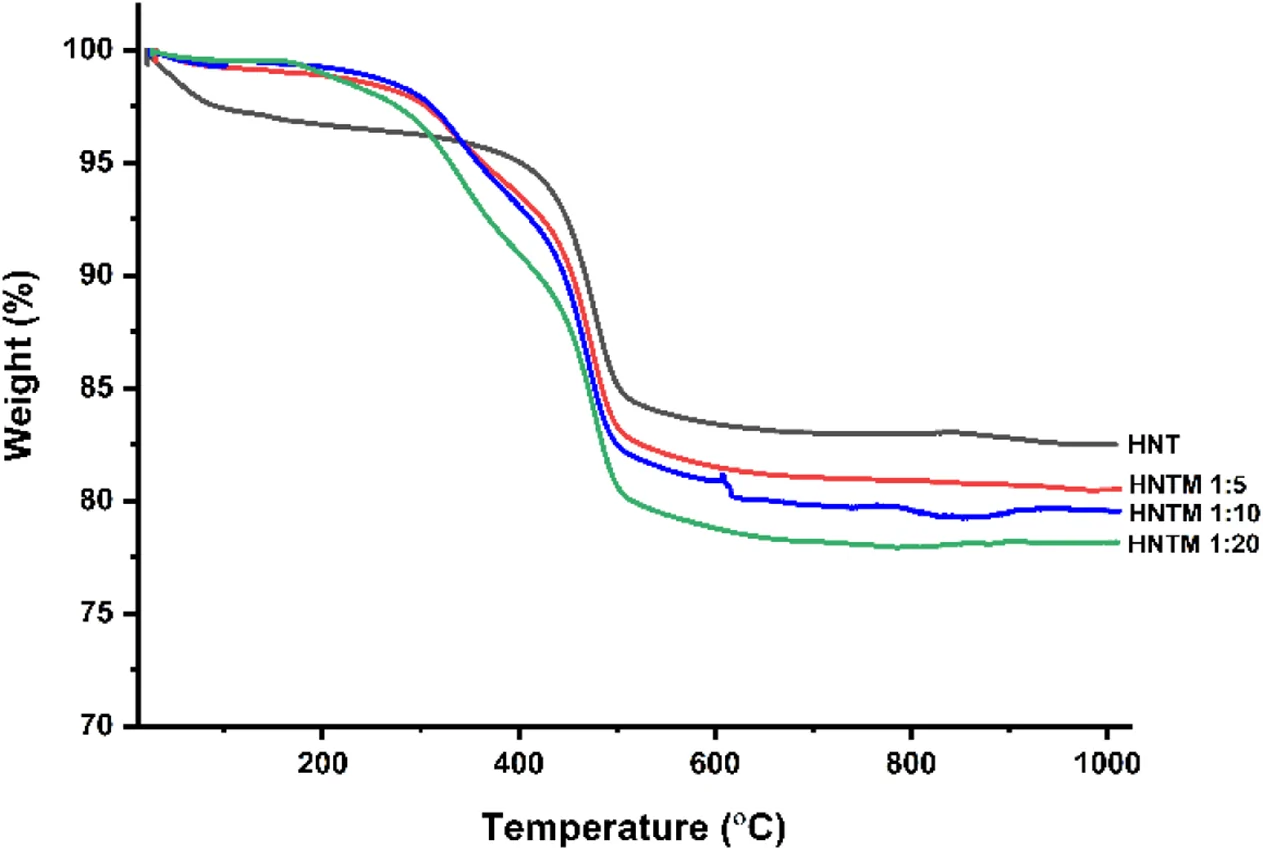
TGA thermograms of HNT and HNTMs.

**Figure 8:**
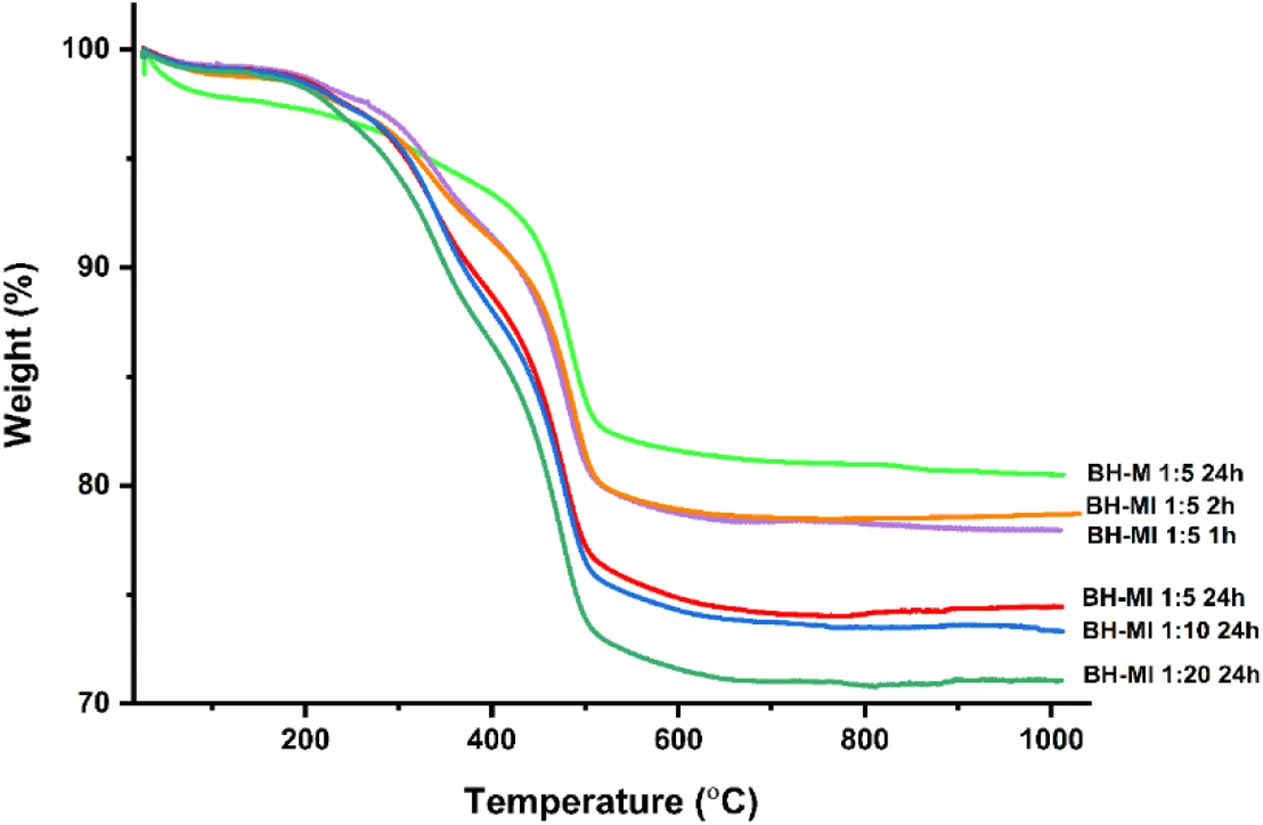
TGA of BH-MIs.

**Figure 9:**
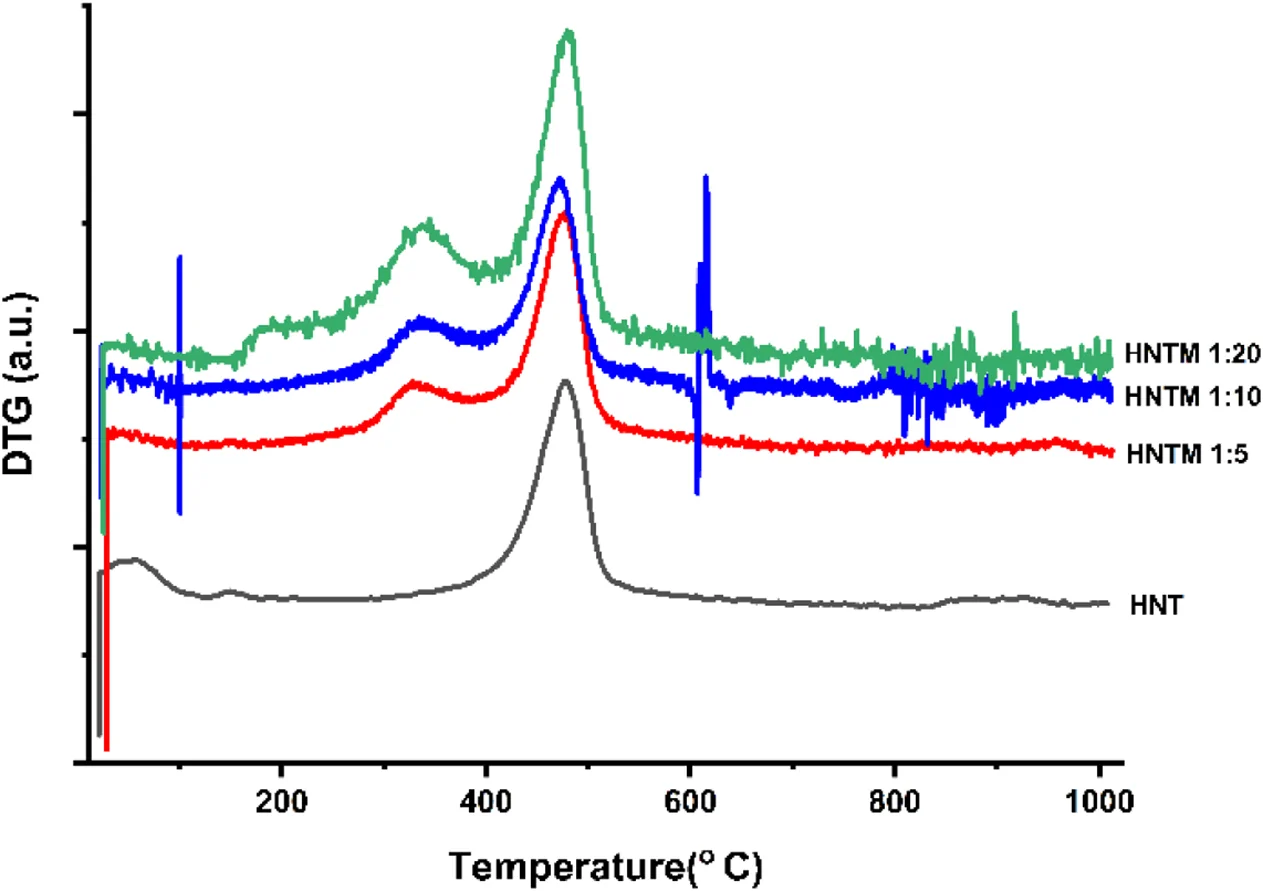
DTG of HNT and HNTMs.

**Figure 10:**
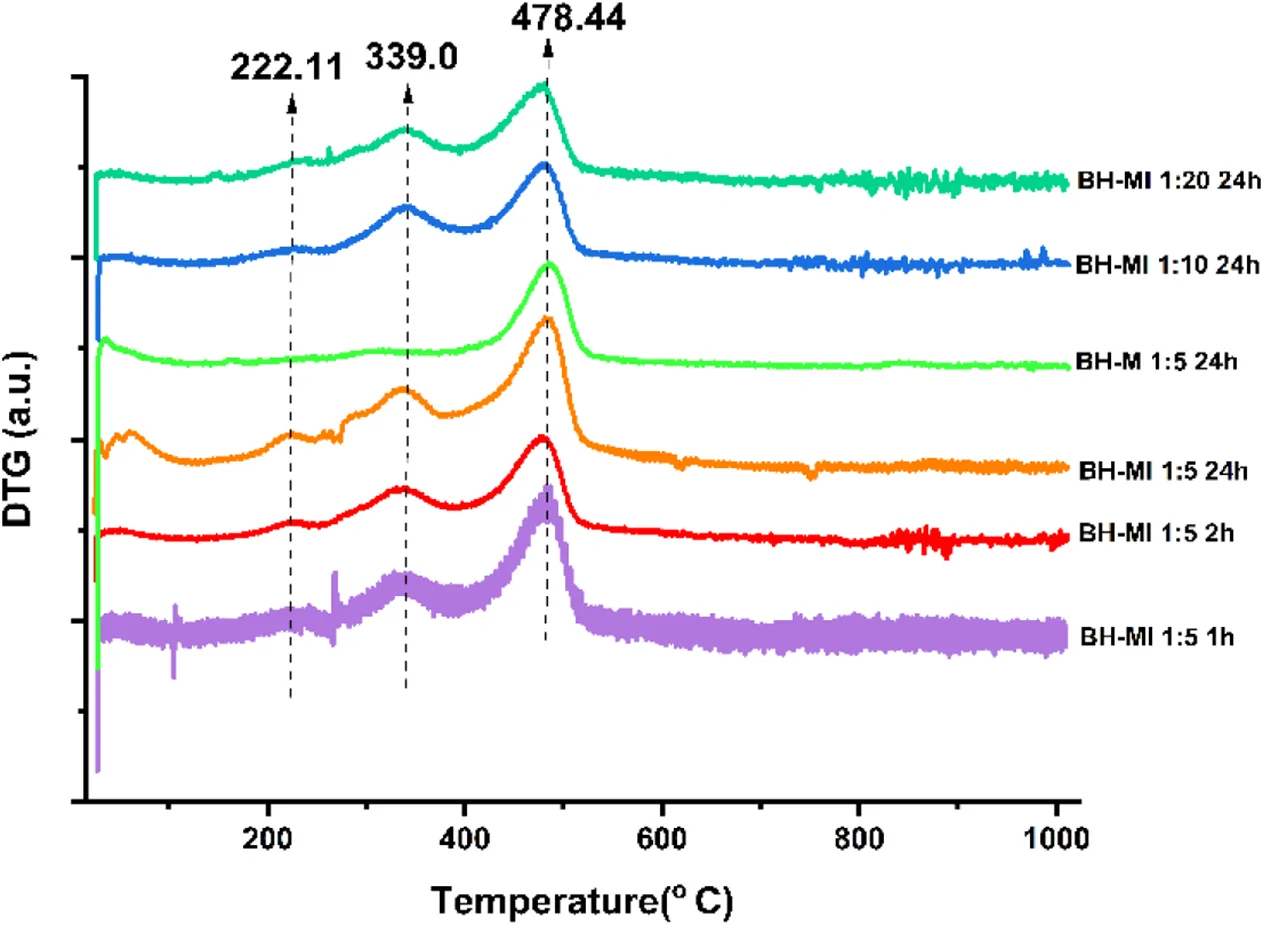
DTG of BH-MIs.

**Figure 11:**
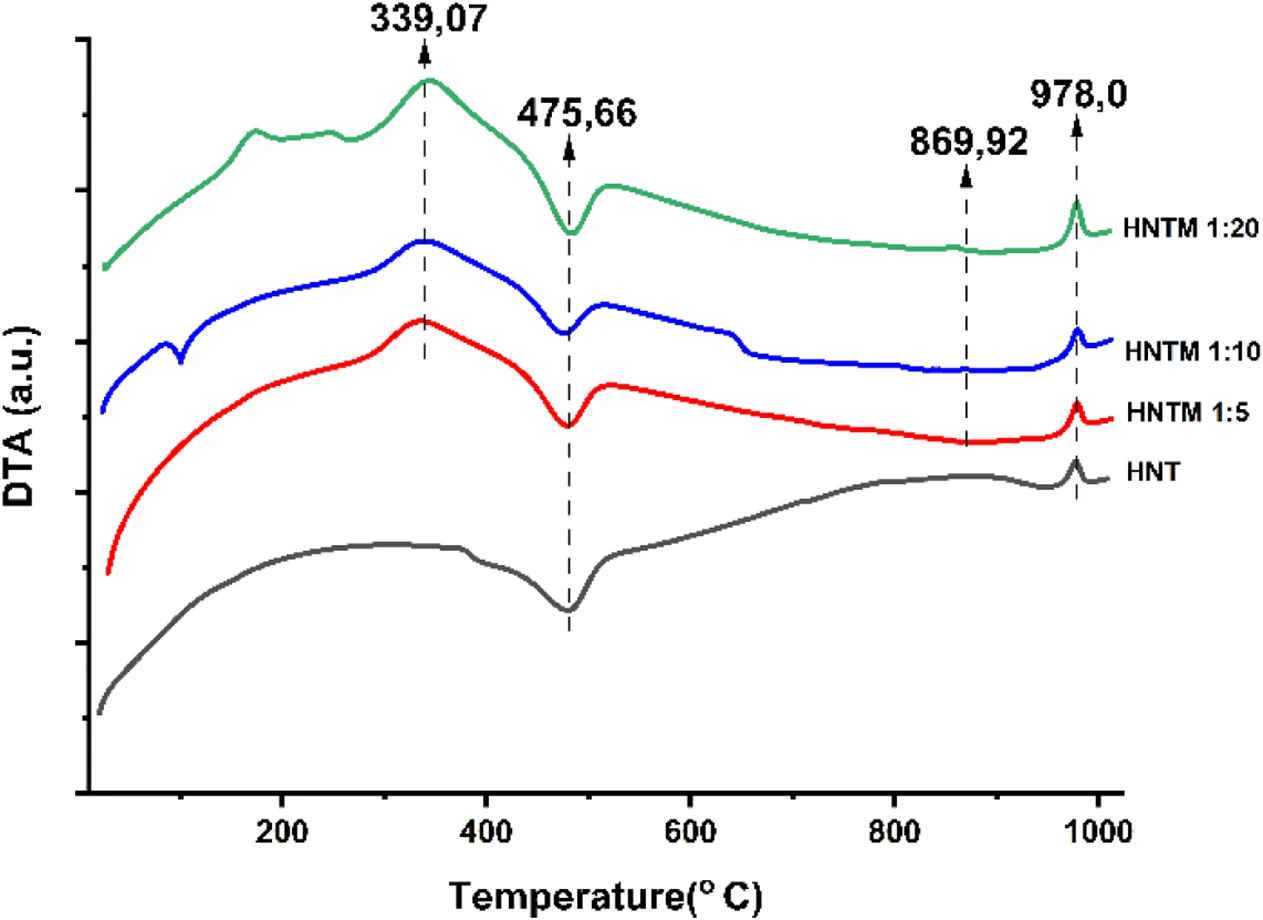
DTA of HNT and HNTMs.

**Figure 12:**
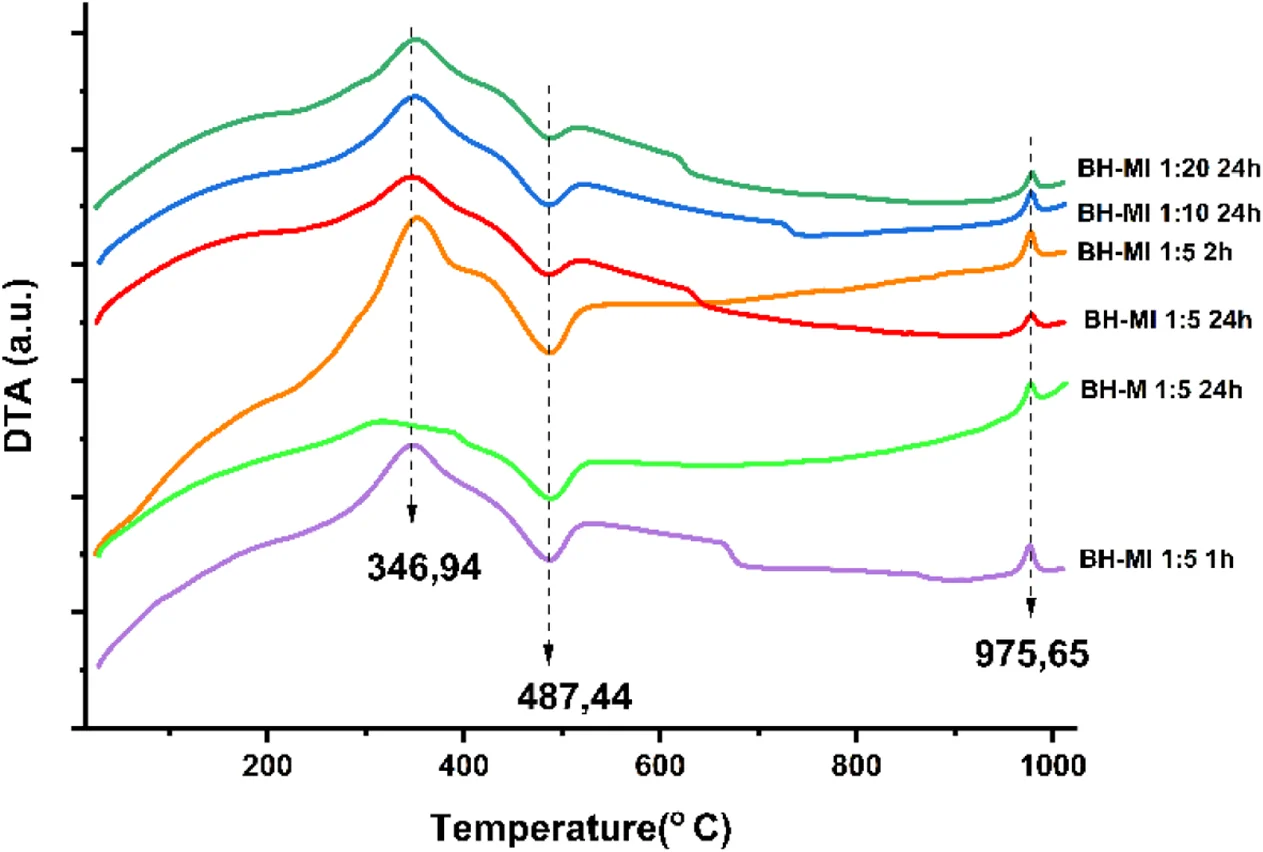
DTA of BH-MIs.

Compared to pure HNT, HNTM samples exhibit a more gradual and multistep mass loss behavior. Notably, an increase in the MAPTES content results in more pronounced mass loss and a higher total mass loss. After excluding the initial mass loss associated with physically bound/adsorbed water, the MAPTES-modified HNT samples exhibited an earlier onset of mass loss than pristine HNT. This behavior can be attributed to the thermal decomposition of the organic MAPTES moieties introduced onto the HNT surface, which occurs at lower temperatures than the major dehydroxylation process of pristine HNT. Nevertheless, the HNTM samples retained the majority of their mass up to approximately 300 °C, indicating that substantial thermal degradation of the modified materials did not occur below this temperature. The small mass variations observed in the low-temperature region were not assigned to a specific MAPTES-related degradation process because no systematic trend with MAPTES loading was observed. The small upward deviation observed near 100 °C was not interpreted as a true mass-gain event. Such minor apparent changes may arise from instrumental baseline effects, including buoyancy and gas-convection effects during heating, and were therefore excluded from the quantitative interpretation.

The changes observed in the 250–400 °C region may reflect overlapping contributions from the thermal decomposition of surface-bound organic species and dehydroxylation processes, with their relative contributions varying with MAPTES content.^48,54^ In addition, in the TGA curve, these mass losses correspond to the DTG peak at about 324 °C (Figure 9). Compared with pure HNT (14.23 wt %), the total mass loss increased to 18.22, 19.61, and 21.57 wt % for HNTM 1:5, HNTM 1:10, and HNTM 1:20, respectively. Accordingly, the silanization yield estimated from the additional mass loss relative to pure HNT increased from 3.99 wt % for HNTM 1:5 and 5.38 wt % for HNTM 1:10 and reached 7.34 wt % for HNTM 1:20. Collectively, these results support the successful incorporation of MAPTES onto the HNT surfaces. Higher silane agent binding could have been achieved in this study; however, Carli et al. related this to the steric hindrance from MAPTES structure. This steric hindrance has been reported to limit the extent of surface modification and may contribute to the relatively low grafting efficiency observed.^64^

Having confirmed successful MAPTES functionalization of HNT, the next step was to evaluate the effect of subsequent PAA polymerization on the coated organic content. In the first series (BH-MI 1:5; 1, 2, and 24 h), the MAPTES ratio 1:5 was kept constant while the polymerization time was varied. In the second series (BH-M 1:5, BH-MI 1:5, BH-MI 1:10, and BH-MI 1:20; all polymerized for 24 h), the polymerization time was fixed while the MAPTES content was systematically varied. The onset of the additional mass loss observed around 200 °C is consistent with the thermal degradation of the coated PAA-containing organic layer on the HNT surface (Figure 8). In the TGA graph, the mass loss of polymeric chains in the composites, whose thermal stability changed after polymerization, started from 200 °C. These mass losses attributed primarily to the thermal degradation of the PAA-containing organic layer correspond to the endothermic peak.

In addition, the endothermic peak has broadened with increasing overall organic coating content, and the DTG peak associated with the MAPTES-containing organic fraction shifted to approximately 339 °C. Consistent with literature reports, the main degradation stages of PAA typically occurred within the 250–400 °C temperature range.^49,67^ A subsequent mass loss occurring between 400 and 500 °C in TGA is associated primarily with the dehydroxylation of structural hydroxyl groups within the HNT framework, with possible contributions from the thermal degradation of the remaining organic phase.^68^ Compared with pure HNT, the additional organic mass loss increased in all PAA-modified samples. This increase is consistent with a higher organic coating content following PAA polymerization. It should be noted that TGA-derived organic mass does not provide a direct measure of PAA molecular weight, chain length, or polydispersity. Therefore, the observed increase in organic mass should be interpreted as an overall increase in grafted/associated organic coating rather than as evidence of longer or more uniform PAA chains.

First, at a fixed MAPTES ratio of 1:5, extending the polymerization time from 1 to 24 h increased the additional coated organic content from 6.65 wt % to 10.60 wt %, although the 2 h sample (6.13 wt %) exhibited a slightly lower value than the 1 h sample. Notably, immobilization with BP increased the additional organic content from approximately 5 wt % in BH-M 1:5–24 h to 10.60 wt % in BH-MI 1:5–24 h, corresponding to an approximately 2-fold increase. Second, when the polymerization time was fixed at 24 h with immobilization of BP, increasing the MAPTES ratio from 1:5 to 1:10 and 1:20 resulted in additional mass losses of 10.60, 11.38, and 13.88 wt %, respectively. As both the reaction time of PAA and MAPTES ratio increased and also immobilized BP, the total mass loss was observed to increase as expected, with the highest overall organic coating content observed for the BH-MI 1:20–24 h sample.

In pure HNT and HNTMs, no mass loss occurs after 500 °C, and only HNT residues are observed; after polymerization, this stability begins after 600 °C in TGA. The difference in mass loss observed in BH-MI samples after 500 °C has been reported as the thermal decomposition of the PAA backbone and the decomposition of the carboxyl groups of the chains.^67^ The additional mass loss observed in the BH-MI samples at higher temperatures may be associated with further thermal decomposition of the PAA-containing organic phase, including decarboxylation and degradation of the polymer backbone.^61^ In the DTA graph, the HNT peak located at 476 °C shifted to 487 °C after polymerization, and the exothermic peak intensity decreased. Although a study conducted by Duce et al. reported that approximately 1% mass loss may occur after 700 °C in HNT clay due to the thermal decomposition of sulfur impurities or alunite, no comparable mass loss was observed in our samples.^69^ In DTA graphs, at 978 °C, the exothermic peak detected could be attributed to the phase transformation of amorphous SiO_2_ and γ-alumina (γ-Al_2_O_3_).

Overall, the TGA, DTG, and DTA analyses demonstrated a progressive increase in the coated organic content with increasing MAPTES ratio and polymerization time. Between 500 and 1000 °C, the HNT residue accounted for 85–70% of the total mass in both graphs.^66^ HNTM 1:20 and the corresponding BH-MI 1:20–24 h samples exhibited the highest total mass loss. When the polymerization time was kept constant (24 h), increasing the MAPTES ratio resulted in progressively higher organic content. Likewise, when the MAPTES ratio was fixed (1:5), extending the polymerization time led to an increase in the overall organic coating content. These findings demonstrate that MAPTES content and polymerization time independently contributed to the increase in the organic coating on the HNT surface. Compared with pure HNT, the total mass loss increased from 14.23% to 21.57% after MAPTES silanization and further increased to 27.11% for the BH-MI 1:20–24 h sample following PAA polymerization. Furthermore, the immobilization of BP further enhanced its effectiveness. Collectively, these thermal analyses confirm the successful surface modification of HNTs and demonstrate that the overall organic coating content can be modulated by varying the MAPTES content and polymerization time.

It is important to note that the TGA-derived increase in the organic mass cannot be assigned to a single molecular parameter. In free-radical photopolymerization, changes in the polymerization time and initiator-mediated reaction conditions may simultaneously affect the number of grafted chains, chain growth, molecular weight, and polydispersity. Because the molecular weight and polydispersity of the surface-bound PAA could not be directly determined in the present system, the TGA results are interpreted here as changes in the overall organic-coating content. Accordingly, the observed increase in organic mass should not be taken as direct evidence of increased chain length or grafting density alone.

TGA, DTG, and DTA results are in good agreement with the FTIR spectra, showing increased characteristic MAPTES silanization and PAA absorption bands, and the SEM images reveal a progressively rougher surface morphology after functionalizations, supporting the formation of an increased organic coating content on the HNT surface.

### Hemostatic Activity of Samples

#### In Vitro Blood Clotting Tests

Activation of coagulation factors is an important parameter in evaluating the materials intended for hemostatic applications. In accordance with the previous results, these experiments were designed to investigate the effects of silanization and differences in the overall PAA-containing organic coating on the coagulation-related behavior of the HNTs. PT, International Normalized Ratio (INR), and aPTT results are presented in Figure 13a and 13b. PT and INR values of the pooled plasma controls were within the normal physiological range.^70–72^

**Figure 13:**
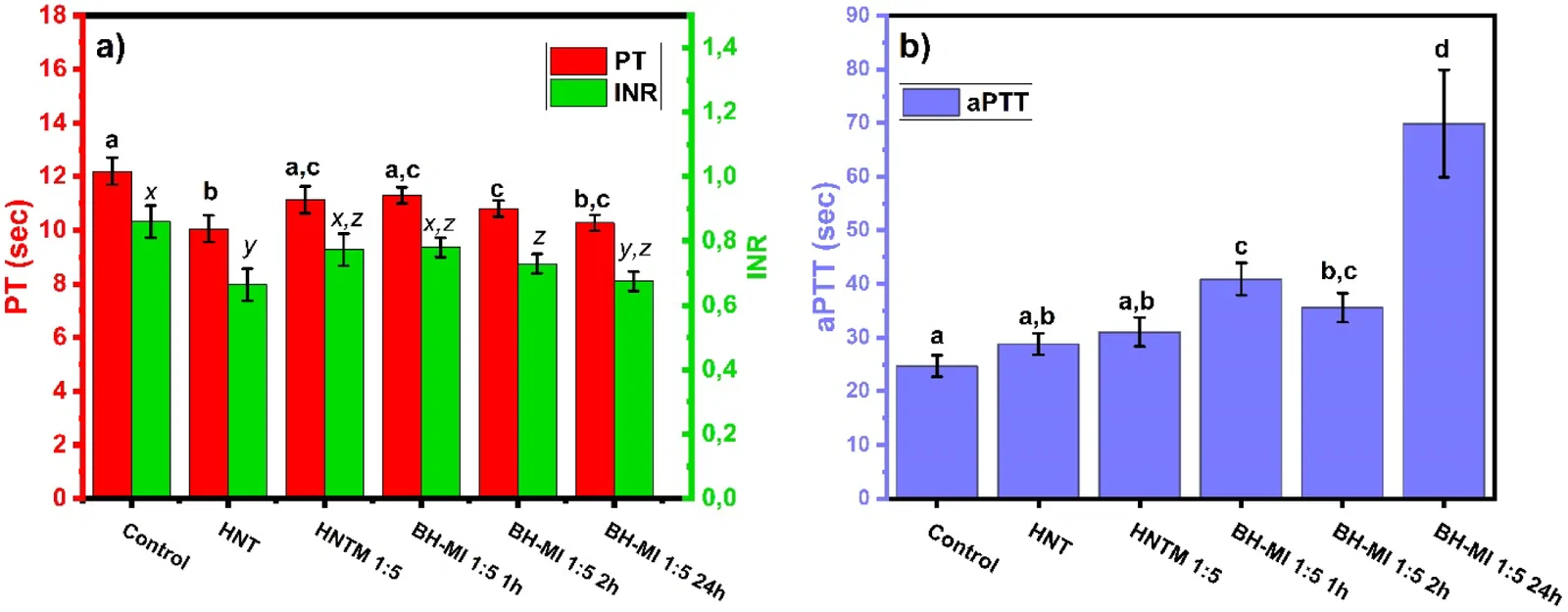
*In vitro* blood clotting tests : a) PT and INR and b) aPTT values of samples. Data are presented as mean ± standard deviation (n = 3). Statistical differences among samples for each parameter were evaluated by one-way ANOVA. Different letters (a-c for PT, x-z for INR, and a-d for aPTT) indicate statistically significant differences between groups (p < 0.05), whereas groups sharing the same letter are not significantly different.

The beneficial effects and rapid action of clay minerals, such as HNT, in wound healing have been previously reported. Owing to their reported hemostatic properties, these materials have been investigated for wound treatment applications.^73^ The observed PT shortening in the HNT sample may be related to several mechanisms, including surface-mediated interactions with coagulation proteins and possible release of Si- and Al-containing species from HNT, as suggested in previous studies.^74,75^ The possible release of Si- and Al-containing species from HNT enables the concentration of blood components through capillary absorption in the liquid-phase environment and provides effective support against thrombosis.^75^ Silanization partially attenuated the PT-shortening effect of unmodified HNT, possibly because MAPTES masked native surface sites involved in plasma–material interactions. Thus, silanization should be regarded mainly as a chemical anchoring step for subsequent polymer modification. Following PAA modification, increasing overall organic coating content was associated with decreased PT and INR values, whereas aPTT values were prolonged in both HNT and PAA-modified HNT samples relative to the control. Similar differences between PT and aPTT responses have also been reported in previous studies, indicating that changes in these two assays do not necessarily follow the same trend.^76^

For example, Song et al. reported the development of cross-linked carrageenan/PAA composite beads, in which increasing PAA content and composite concentration resulted in prolonged aPTT values.^77^ Similarly, An et al. reported that membranes prepared from PVDF-*g*-(acryloylmorpholine) and PVDF-*g*-(poly­(acrylic acid)-argatroban) copolymers exhibited prolonged aPTT values following PAA modification. Furthermore, incorporation of PAA increased surface hydrophilicity and improved water permeability, antifouling performance, and blood compatibility of the membranes.^78^

PAA-modified HNTs showed different effects on PT and aPTT, with shortening of PT and prolongation of aPTT compared to control. These findings indicate that the materials affected the two coagulation assays differently. However, PT and aPTT alone cannot establish selective activation of the extrinsic pathway or inhibition of the intrinsic pathway. Because PT and aPTT are reagent-dependent plasma assays, the observed changes may arise from interactions between the material surface and plasma components or other assay-related effects. Indeed, certain studies have reported that changes in PT and aPTT do not necessarily follow the same trend, with one parameter remaining unchanged while the other varies.^76^ Overall, these results indicate that PAA-modified HNTs warrant further investigation as candidate materials for hemostatic applications.

#### Platelet Adhesion Test

Platelet adhesion results of samples are presented in Figure 14. The platelet results demonstrated that exposure to pure HNT resulted in nearly complete depletion of the recoverable platelet fraction (≈99%) at the initial time point, suggesting a pronounced interaction with the recoverable platelet fraction. The PAA-modified HNT samples showed different *in vitro* coagulation and platelet-recovery profiles compared with unmodified HNTs. HNTs have positively charged Al–OH groups on their inner surfaces and negatively charged Si–O–Si siloxane groups on their outer surfaces.^15^ In addition, HNTs exhibit superhydrophilic behavior, with a water contact angle of approximately 10°, and thus, upon contact with blood, they may absorb substantial amounts of water, leading to local concentration of blood components and potentially contributing to hemolytic effects at high HNT concentrations.^79,80^ The MAPTES-silanized HNT sample showed a slight reduction in platelet adhesion. Surface modification of HNTs with MAPTES introduces organic methacrylate chains that increase surface hydrophobicity, thereby potentially reducing protein and platelet adsorption.^81^ The MAPTES coating alters the physicochemical characteristics of the HNT surface and may reduce direct interactions between the material and blood components. Accordingly, MAPTES-silanized HNTs exhibited lower platelet depletion than unmodified HNTs. However, the specific contribution of surface chemistry to this behavior cannot be determined from the present data.

**Figure 14:**
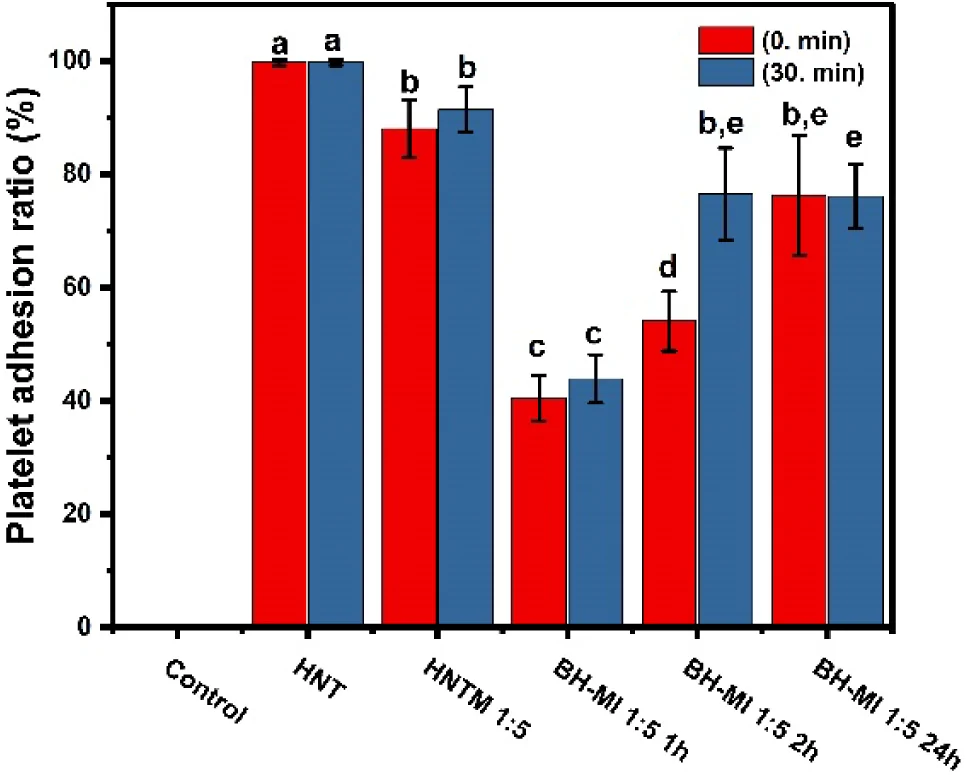
Platelet adhesion results of samples. Data are expressed as mean ± SD (n = 3). Statistical differences among samples were assessed separately at each time point (0 and 30 min) by one-way ANOVA followed by Tukey's post hoc test. Different letters indicate statistically significant differences (p < 0.05), whereas samples sharing at least one letter are not significantly different. No platelet adhesion was observed in the Control group.

In contrast, PAA-modified HNT samples exhibited more moderate platelet adhesion behavior. Notably, the BH-MI 1:5 2 h sample showed a pronounced increase in platelet adhesion from 54% to 76% within 30 min, suggesting an increased interaction between the material and the recoverable platelet fraction. Similarly, the BH-MI 1:5 1 h sample exhibited a modest increase from 40% to 44%. The decrease in measurable platelet concentration following material exposure was interpreted as platelet depletion from the recoverable PRP fraction. Because this method cannot distinguish surface adhesion, aggregation, physical entrapment, sedimentation, or analyzer-related effects, it should not be considered a direct measure of platelet activation. Unmodified HNT produced the greatest reduction in the recoverable platelet count, whereas PAA modification altered the magnitude of this effect. Therefore, the observed platelet adhesion values should be interpreted as an indication of platelet–material interaction rather than as direct evidence of enhanced or impaired hemostatic performance. The relatively low platelet depletion observed for this sample may reflect differences in surface coverage or particle behavior; however, the available data are insufficient to distinguish among these possibilities. In a recent study by Zhang et al., thermal polyurethane (TPU) catheters were modified with polyacrylamide (PAM)/PAA-based polymer brushes, resulting in a marked improvement in surface hemocompatibility.^82^ This enhancement was primarily associated with increased surface hydrophilicity and the formation of a stable hydration layer, which may reduce direct interactions between blood components and the material surface. Overall, the present findings suggest that PAA modification alters the platelet–HNT interaction and modulates the extent of platelet adhesion. However, further analyses, such as platelet activation markers, platelet morphology, or complementary hemocompatibility assays, would be required to determine whether these changes correspond to beneficial hemostatic activity or undesirable platelet activation.

## Conclusion

In this study, PAA chains were successfully incorporated onto MAPTES-silanized HNTs via photoinduced polymerization to develop novel hemostatic biocomposites. Comprehensive characterization (FT-IR, TGA, zeta potential, XRD, and SEM) supported the successful surface modification of HNTs with MAPTES and PAA while confirming preservation of the characteristic HNT tubular structure. The results demonstrated that the overall organic coating content could be modulated by varying the polymerization time and MAPTES ratio. Because the molecular weight, chain length, and polydispersity of the surface-grafted PAA were not directly measured, the effects observed here should be interpreted as resulting from changes in the overall organic coating rather than from independently resolved changes in grafting density or polymer chain length.

Initial *in vitro* biological evaluations revealed that the PAA-coated HNTs possess promising hemostatic potential. Plasma coagulation assays showed that the modified nanocomposites altered both PT and aPTT responses relative to pristine HNTs, with the magnitude of these changes varying with the overall organic coating content. Furthermore, PAA modification altered the extent of platelet depletion from the recoverable plasma fraction compared with pristine HNTs. These findings suggest that PAA modification influences platelet–material interactions; however, further studies are required to determine whether these changes are associated with improved hemocompatibility or platelet activation.

Importantly, the biological evaluations in the present study were performed using the 1:5 MAPTES ratio, while the 1:10 and 1:20 MAPTES ratios were characterized primarily in terms of their physicochemical and thermal properties. Therefore, the biological effects observed here should not be interpreted as evidence of hemostatic tunability across different MAPTES ratios. Within the evaluated 1:5 series, variation in the polymerization time resulted in differences in the overall organic coating content and was associated with changes in the measured coagulation and platelet-recovery responses.

Overall, PAA modification of HNTs provides a surface-modification strategy with tunable overall organic coating content, particularly through variation of polymerization time, and promising hemostatic characteristics, which warrant further investigation through whole-blood and *in vivo* studies.

## Data Availability

The raw/processed data required to reproduce these findings cannot be shared at this time, as the data also forms part of an ongoing study.
